# Inhibition of New Delhi Metallo-β-Lactamase 1 (NDM-1) Producing *Escherichia coli* IR-6 by Selected Plant Extracts and Their Synergistic Actions with Antibiotics

**DOI:** 10.3389/fmicb.2017.01580

**Published:** 2017-08-22

**Authors:** Brinda Chandar, Sundar Poovitha, Kaliappan Ilango, Ramasamy MohanKumar, Madasamy Parani

**Affiliations:** ^1^Genomics Laboratory, Department of Genetic Engineering, School of Bioengineering, SRM University Kattankulathur, India; ^2^Interdisciplinary Institute of Indian System of Medicine, SRM University Kattankulathur, India

**Keywords:** antibacterial activity, antimicrobial resistance, infectious disease, medicinal plants, NDM-1, *Escherichia coli*, phytochemical

## Abstract

Improper use of antibiotics has led to a great concern in the development of pathogenic microbial resistance. New Delhi metallo-β-lactamase 1 (NDM-1) producing bacteria are resistant to most of the β-lactam antibiotics, and so far, no new compounds have been clinically tested against these bacteria. In this study, ethanol extracts from the leaves of 240 medicinal plant species were screened for antibacterial activity against an NDM-1 *Escherichia coli* strain. The extracts that showed antibacterial activity were then tested for minimum inhibitory concentrations (MICs) and zones of inhibition. The extract from *Combretum albidum* G. Don, *Hibiscus acetosella* Welw. ex Hiern, *Hibiscus cannabinus* L., *Hibiscus furcatus* Willd., *Punica granatum* L., and *Tamarindus indica* L. showed bactericidal activity between 5 and 15 mg/ml and the MIC was between 2.56 and 5.12 mg/ml. All six plant extracts inhibited activity of the NDM-1 enzyme *in vitro*, and the IC50 value ranged between 0.50 and 1.2 ng/μl. Disruption of bacterial cell wall integrity by the plant extracts was clearly visible with scanning electron microscopy. Increases in membrane permeability caused 79.4–89.7% bacterial cell deaths as investigated by fluorescence-activated cell sorting. All the plant extracts showed synergistic effects when combined with colistin [fractional inhibitory concentration (ΣFIC) = 0.125–0.375], meropenem (ΣFIC = 0.09–0.313), and tetracycline (ΣFIC = 0.125–0.313). Thus, the plant extracts can be fractionated for the identification of active compounds, which could be used as new antibacterial compounds for the development of drugs against NDM-1 *E. coli* in addition to their use in combination therapy.

## Introduction

Extensive use of antibiotics in the past has led to the emergence of bacterial resistance among pathogenic microorganisms. Bacteria are known for their ability to adapt to the environment, and evolve into antibiotic-resistant forms by modifying their genetic makeups (Toleman et al., 2006). Production of detoxifying enzymes, efflux pumps, and altered receptor sites for antibiotics are some ways in which they acquire resistance. β-Lactam antibiotics account for about 60% of all antibacterial agents used to treat the infections caused by Gram-negative bacteria (Livermore and Woodford, 2006). Bacteria counteract these antibiotics by acquiring the ability to produce β-lactamases, extended spectrum β-lactamases, AmpC enzymes, and metallo-β-lactamases (MBLs) (Carattoli, 2009). Bacterial capability to acquire MBLs and become difficult-to-treat “superbugs” is significant (Toleman et al., 2006; Patzer et al., 2009).

The recently discovered New Delhi metallo-β-lactamase 1 (NDM-1), which confers extensive antibiotic resistance against most of the currently available β-lactam antibiotics, is a global concern. New Delhi metallo-β-lactamase 1 was first identified in *Klebsiella pneumoniae* and *Escherichia coli* from New Delhi, and subsequently reported in 11 other bacterial species from several countries including Austria, Australia, Bangladesh, Canada, China, Europe, Hong Kong, Japan, Kenya, Oman, Pakistan, Singapore, United States, and Taiwan (Yong et al., 2009; Hammerum et al., 2010; Bhaskar, 2012). Although NDM-1 was initially believed to be acquired in hospitals, later studies reported the presence of NDM-1 bacteria in environmental samples as well (Walsh et al., 2011; Isozumi et al., 2012; Wang and Sun, 2015). Several NDM isoforms (NDM-2 to NDM-7) with variations in antibiotic susceptibility profiles were also reported (Hornsey et al., 2011; Góttig et al., 2013). Evolution of new resistance patterns in several bacteria is continuous; hence, the World Health Organization focuses on control, and prevention of overuse and misuse of antibiotics. However, the plasmid-borne nature of NDM-1 facilitates its rapid dissemination within and beyond Enterobacteriaceae (Norman et al., 2009). Therefore, it is necessary to explore the possibility of finding new and effective antibacterial compounds against NDM-1 bacteria.

The NDM-1 enzyme has two zinc ions in its active site, which are essential for cleaving the C–N bond in order to inactivate the β-lactam antibiotics. Compared to other MBLs, lysine-rich NDM-1 is considered more favorable for protonation of lactam nitrogen, which may contribute to its resistance against a wide range of β-lactam antibiotics (Liang et al., 2011). So far, only a few potential molecules have been identified to have the capability to inhibit NDM-1 enzyme activity. Guo et al. (2011) have reported that D-captopril binds to the active site of recombinant NDM-1 with high binding affinity and inhibits its enzymatic activity. Aspergillomarasmine A (AMA), a natural compound from *Aspergillus versicolor*, was reported to inhibit NDM-1 activity by extracting the zinc ions from its active site. Aspergillomarasmine A was able to fully restore the antibacterial activity of meropenem against NDM-1 producing Enterobacteriaceae, *Acinetobacter* spp. and *Pseudomonas* spp. (King et al., 2014). Ebselen was shown to covalently bind with the cysteine residue at the active site of NDM-1 thus inhibiting its activity. However, toxicity of the selenium moiety in ebselen may limit its potential as prospective drug against NDM-1 bacteria (Chiou et al., 2015).

Although efforts have been made to discover advanced strategies to combat NDM-1 resistance, determination of activity against such bacteria using medicinal plants is limited. Standardized extracts from plants can provide new and safe antibacterial drugs because of the high chemical diversity present in them. Although 65% of the antibacterial drugs approved between the years 1981 and 2010 were natural products or their semi-synthetic derivatives (Newman and Cragg, 2012), they still remain to be investigated in order to find molecules that are effective against NDM-1 bacteria. Against this background, 240 taxonomically diverse set of medicinal plants species from 183 genera and 75 families were investigated, and ethanol extracts from the leaves of six species were identified as potential sources for antimicrobial compounds against NDM-1 bacteria. In addition, studies were carried out in order to understand the mechanism of action of the extracts and their combinatory effects with selected antibiotics.

## Materials and Methods

### NDM-1 *Escherichia coli*

A clinical isolate of NDM-1 *E. coli* strain used in this study was a generous gift from Dr. David M. Livermore, Health Protection Agency Centre for Infections, London, United Kingdom (Kumarasamy et al., 2010). Plasmid DNA was isolated according to the protocol described by Sambrook et al. (1989) and a region of NDM-1 gene was amplified using NDM-1 specific primers F-(5′-GGGCAGTCGCTTCCAACGGT-3′) and R-(5′-GTAGTGCTCAGTGTCGGCAT-3′) (Yong et al., 2009; Manchanda et al., 2011). 16S rDNA was amplified by colony PCR using the universal forward and reverse primers (5′-GAGTTTGATCATGGCTCAG-3′) and (5′-CTACGGCTACCTTGTTACG-3′), respectively (Grifoni et al., 1995). Sequencing of 16S rRNA and NDM-1 gene was done for confirmation of the strain. New Delhi metallo-β-lactamase 1 *E. coli* resistance to different antibiotics was determined according to the performance standards for antimicrobial disk susceptibility testing given by Clinical and Laboratory Standards Institute for *E. coli* (Clinical and Laboratory Standards Institute, 2014). The presence of MBL activity was tested using the double disk synergy test in the presence of ethylenediaminetetraacetic acid (EDTA) as the chelating agent and meropenem as the β-lactam antibiotic as previously described by Arakawa et al. (2000).

### Collection of Plant Material

Two hundred and forty species from 183 genera and 75 families, listed as medicinal plants by the National Medicinal Plants Board of India, were selected. Leaf samples were collected from different parts of Southern India including Karnataka, Kerala, and Tamil Nadu. Voucher specimens from all the collections were identified using local floras and were deposited in the SRM University Herbarium (Supplementary Table S1).

### Preparation of Extracts

Fresh mature leaves were thoroughly rinsed with running water followed by 70% ethanol, dried, and stored at room temperature. The dried leaves were ground to a powder in a laboratory blender, labeled, and stored in air tight containers at room temperature until use. The powder (10 g) was mixed with 100 ml of ethanol, and the mixture was macerated in a shaker incubator at 37°C and 150°*g* for 24 h. This process was repeated twice, and the macerated samples were filtered through Whatman No. 1 filter paper. Residual solvent from the extracts was removed using a rotary evaporator under reduced pressure, and stored at 4°C.

### Screening of Plant Extracts against NDM-1 *E. coli*

The plant extracts were dissolved in dimethyl sulfoxide (DMSO), and screened for bactericidal activity against an NDM-1 *E. coli* strain. The cells from the agar slants were recovered by inoculating into nutrient broth containing 100 μg/ml cefotaxime, for the maintenance of plasmid-borne NDM-1 gene (Kumarasamy et al., 2010). The culture was incubated at 37°C and 150 *g* until its growth reached 0.5 McFarland standard (10^8^ cfu/ml). Plant extract was added to yield final concentrations of 5–25 mg/ml (in 5 mg increments) and inoculated with 100 μl of NDM-1 *E. coli* culture. Dimethyl sulfoxide (4%) was used as a negative control. The cultures were grown in a shaker incubator at 37°C and 150 *g* for 24 h. The cultured cells were streaked on MHA plates, incubated at 37°C for 16 h, and observed for bacterial growth.

### Determination of Minimum Inhibitory Concentration

Minimum inhibitory concentration (MIC) was determined using the Resazurin Microplate Assay as described by Palomino et al. (2002) with minor modifications. Stock solutions of extracts were prepared in DMSO and NDM-1 *E. coli* cells were grown to 0.5 McFarland standard. Serial dilutions were made by microbroth dilution technique, and the total volume was brought up to 100 μl with Muller Hinton broth. Colistin and DMSO were used as positive and negative controls, respectively. Sealed microtiter plates were incubated overnight at 37°C. After the incubation period, 3.0 μl of 0.03% resazurin was added to the wells as an indicator of cell viability, and then incubated at 37°C for 4 h.

### Determination of the Zone of Inhibition

Antibacterial activity of plant extracts was determined by measuring the zones of inhibition using the disk diffusion method (Bauer et al., 1966). Bacterial cells were grown to 0.5 McFarland standard, swabbed on Mueller Hinton Agar (MHA) media, and allowed to dry for 10 min. The disks loaded with 5–20 mg (in 5 mg increments) of plant extracts were then placed on the MHA plates. The disks loaded with colistin (10 μg/disk) and DMSO (20 μl/disk) were used as positive and negative controls, respectively. The culture plates were incubated at 37°C for 24 h, and the zones of inhibition were measured in triplicate for each plant extract.

### Checkerboard Synergy Testing

Synergy testing of plant extracts and three antibiotics was performed in 96-well microtiter plates by following the checkerboard method (Bayer and Morrison, 1984). New Delhi metallo-β-lactamase 1 *E. coli* cells were inoculated in nutrient broth and incubated at 37°C for 16 h. Combinations of plant extract (10–5120 μg/ml) with colistin (0.5–8 μg/ml), meropenem (0.5–32 μg/ml), and tetracycline (1–16 μg/ml) were tested. Interactions between the antibacterial agents were determined by calculating the fractional inhibitory concentration (ΣFIC) index:

$$\Sigma FIC=\frac{\mathrm{MIC}\;E+D}{\mathrm{MIC}\;E}+\frac{\mathrm{MIC}\;E+D}{\mathrm{MIC}\;E}$$

where MIC E+D is the MIC of extract in combination with antibiotic and MIC D+E is the MIC of antibiotic in combination with extract. Based on the ΣFIC, interaction between the antibiotic and the extract was inferred to have a synergistic (ΣFIC < 0.5), additive (0.5 > ΣFIC < 1), indifferent (1 > ΣFIC < 4), or antagonist (ΣFIC > 4) effects (dos Santos et al., 2015).

### NDM-1 Enzyme Inhibition Assay

The NDM-1 enzyme inhibition assay was carried out using nitrocefin as a substrate as described by King et al. (2014) with minor modifications. Nitrocefin and the plant extracts were dissolved in DMSO. In a microplate, 5 nM recombinant NDM-1 enzyme (Cusa Bio, China) supplemented with 10 μM ZnSO_4_ was incubated with plant extract (0.02–5.12 ng/μl) or 10 μl of DMSO as negative control for 10 min. After the incubation, nitrocefin was added to a final concentration of 60 μM, and absorbance was measured at 490 nm and 30°C temperature using a multi-mode reader (BioTek Synergy, United States). The percentage inhibition was estimated, and IC50 values were determined for each extract.

### Flow Cytometry

Membrane integrity of the NDM-1 *E. coli* cells after treatment with plant extracts was analyzed as described by Kennedy et al. (2011) using the LIVE/DEAD^®^ BacLight^TM^ Kit (Thermo Scientific, United States). Flow cytometry was optimized by preparing a standard by mixing live and dead cells in phosphate buffer. Bacterial cell density in the mid-exponential growth phase was adjusted to 1 × 10^8^ cfu/ml, and treated with 2× MIC of plant extracts at 37°C for 1 h. The treated bacterial cells were incubated with 5 μM SYTO 9 in the dark for 15 min, and propidium iodide (PI) was added to a final concentration of 30 μM. Colistin (4 μg/ml) and DMSO (4%) were used as positive and negative controls, respectively. The cells were analyzed in a flow cytometer and the signals were captured using FL1 and FL3 channels (BD FACS Calibur, United States).

### Scanning Electron Microscopy

Morphological changes in the NDM-1 *E. coli* cells after treatment with plant extracts were observed following the method described by Hartmann et al. (2010) using a Vega 3 scanning electron microscope (SEM; Tescan, United States). Cells grown in nutrient broth with or without DMSO were used as negative controls and colistin as a positive control. Bacterial cell density in the mid-exponential growth phase was adjusted to 1 × 10^8^ cfu/ml and treated with 2× MIC of plant extracts for 1 and 4 h at 37°C. The cells were centrifuged and the pellet was resuspended in 100 μl of phosphate buffer (pH 7.0). The cell suspension was spread on a glass slide, and fixed using 2.5% glutaraldehyde. The fixed cells were serially washed in ethanol ranging from 10 to 90%, dried, and observed under the microscope.

### Phytochemical Analysis

Qualitative analysis of the phytochemicals present in the plant extracts was carried out following standard protocols (Harborne, 1973). Quantitative assessment of total glycosides, phenolic compounds, saponins, steroids, flavonoids, alkaloids, and terpenoids was carried following the protocols described by Masuko et al. (2005), Zhang et al. (2007), Xu and Chang (2009), Daksha et al. (2010), Herald et al. (2012), Li et al. (2014), and Lin et al. (2015), respectively.

## Results

### Confirmation of the NDM-1 *E. coli* Strain

The NDM-1 *E. coli* IR-6 strain used in this study was confirmed by DNA sequencing, and the antibiotic susceptibility and double disk synergy tests. The size of NDM-1 gene amplified from plasmid DNA was 475 bp and that of the 16S rDNA amplified from genomic DNA was 1500 bp. BLAST analysis of 16S rDNA showed 100% identity with *E. coli*, and that of NDM-1 gene showed 100% identity with the NDM-1 gene reported from many bacteria including *E. coli* (Acc. No. CP021210.1). Antibiotic susceptibility test was conducted using different classes of antibiotics, which included aminoglycosides, cephalosporins, fluoroquinolones, β-lactams, extended-spectrum β-lactams, polymyxins, and carbapenems. The NDM-1 *E. coli* IR-6 strain was susceptible to all the antibiotics tested, except colistin (**Table 1**). There were no zones of inhibition for most of the antibiotics tested indicating high levels of resistance against these antibiotics. In the double disk synergy test, the zone of inhibition for meropenem was 12.6 ± 0.57 mm, and this zone increased to 24.3 ± 1 mm in the presence of EDTA due to chelation of metal ions, which indicated that this strain harbors MBL.

**Table 1:** Resistance or susceptibility of the NDM-1 *E. coli* strain used in the present study to different antibiotics.

		Concentration of the	Resistant/
S. No.	Antibiotics	antibiotic (μg/disk)	susceptible^∗^
1	Amikacin	30	Resistant
2	Ampicillin	10	Resistant
3	Cefoperazone	75	Resistant
4	Cefixime	5	Resistant
5	Cefotaxime	30	Resistant
6	Ceftazidime	30	Resistant
7	Ceftriaxone	30	Resistant
8	Ciprofloxacin	5	Resistant
9	Colistin	10	Susceptible
10	Gentamicin	10	Resistant
11	Imipenem	10	Resistant
12	Meropenem	10	Resistant
13	Ofloxacin	5	Resistant
14	Piperacillin–tazobactam	100/10	Resistant
15	Tetracycline	30	Resistant
16	Tigecycline	15	Resistant
17	Tobramycin	10	Resistant

*^∗^As per the performance standards for antimicrobial disk susceptibility test for *E. coli* ATCC 25922 according to the Clinical and Laboratory Standards Institute (CLSI) guidelines.*

### Antibacterial Activity of the Plant Extracts

Ethanol extract from the leaves of 240 medicinal plants was tested against NDM-1 *E. coli* at concentrations 5–25 mg/ml (in 5 mg increments) to identify extracts that have antibacterial activity. The estimation of minimum bactericidal concentration (MBC) showed antibacterial activity for only 12 medicinal plant extracts against NDM-1 *E. coli*. Among them, the extracts from six plants, which showed MBCs between 5 and 15 mg/ml were taken for further studies. Minimum bactericidal concentration for the extract from *Hibiscus cannabinus* (HC) and *Tamarindus indica* (TI) was 5 mg/ml, *Combretum albidum* (CA) and *Hibiscus acetosella* (HA) was 10 mg/ml, and *Hibiscus furcatus* (HF) and *Punica granatum* (PG) was 15 mg/ml (**Table 2**).

**Table 2:** Ethanol extract from the leaves of 12 medicinal plant species that showed minimum bactericidal concentration (MBC) against NDM-1 *E. coli*.

S. No	Name of the plant	Abbreviation	MBC (mg/ml)^∗^				
			5	10	15	20	25
1	*Albizia saman* (Jacq.) Merr.	AS	-	-	-	-	+
2	*Bixa orellana* L.	BO	-	-	-	-	+
3	*Combretum albidum* G. Don	CA	-	+	+	+	+
4	*Hibiscus acetosella* Welw. ex Hiern	HA	-	+	+	+	+
5	*Hibiscus cannabinus* L.	HC	+	+	+	+	+
6	*Hibiscus furcatus* Willd.	HF	-	-	**+**	+	+
7	*Phyllanthus emblica* L.	PE	-	-	-	-	+
8	*Plumeria rubra* L.	PR	-	-	-	-	+
9	*Punica granatum* L.	PG	-	-	**+**	+	+
10	*Tamarindus indica* L.	TI	+	+	+	+	+
11	*Terminalia muelleri* Benth.	TM	-	-	-	-	+
12	*Vitex altissima* L.f.	VA	-	-	-	-	+

*^∗^Bactericidal activity present (+) or absent (-).*

**FIGURE 1:**
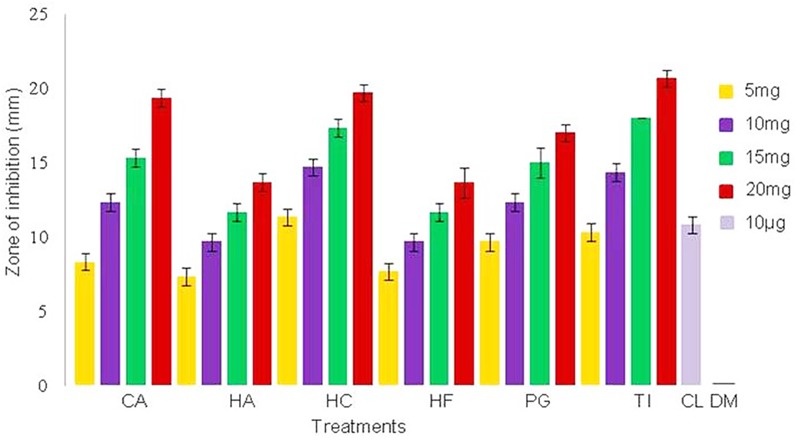
Zone of inhibition of NDM-1 *E. coli* measured in triplicate (mean ± SD) using the disk diffusion method. The disks were loaded with 5, 10, 15, and 20 mg of plant extracts from *Combretum albidum* (CA), *Hibiscus acetosella* (HA), *Hibiscus cannabinus* (HC), *Hibiscus furcatus* (HF), *Punica granatum* (PG), and *Tamarindus indica* (TI). Disks loaded with 10 μg of colistin and 20 μl of DMSO were used as positive and negative controls, respectively. No zone of inhibition was observed in the negative control.

Minimum inhibitory concentrations of the plant extracts against NDM-1 *E. coli* were estimated to be 2.56 mg/ml for CA, HA, HC, and TI, and 5.12 mg/ml for HF and PG. Antimicrobial activity of the extract was also determined by measuring the zone of inhibition of bacterial growth around the disks that were loaded with plant extracts at concentrations of 5–20 mg/disk. Zones of inhibition were observed against NDM-1 *E. coli* at all the concentrations of the plant extracts tested. At the lowest concentration of plant extract tested (5 mg/disk), the HC plant extract showed the highest zone of inhibition (11.6 ± 0.57 mm) followed by the plant extracts of TI (10.3 ± 0.57 mm), PG (9.6 ± 0.57 mm), CA (8.3 ± 0.57 mm), HF (7.6 ± 0.57 mm), and HA (7.3 ± 0.57 mm). *Hibiscus cannabinus* also exhibited the highest zone of inhibition at all the concentrations. Colistin (10 μg/disk), which was used as a positive control, showed a zone of inhibition of 10.8 ± 0.57 mm, and DMSO (20 μl/disk), which was used as a negative control, did not show any zone of inhibition (**Figure 1**).

### Phytochemical Analysis of the Plant Extracts

Phytochemical analysis of these extracts showed variations in the quantity of total phenolic compounds, terpenoids, alkaloids, glycosides, steroids, flavonoids, and saponins (**Table 3**). All of the extracts contained very low (<1.0 μg/ml) to low quantity (1.0–3.0 μg/ml) of steroids and saponins. While CA, HA, HC, and HF contained flavonoids as the major secondary metabolite (7.34 ± 0.02 to 9.69 ± 0.25 μg/ml), PG contained phenolic compounds as the major secondary metabolite (11.02 ± 0.49). Notably, TI contained very low (<1.0 μg/ml) or low quantity (1.0–3.0 μg/ml) of all secondary metabolites that were tested.

**Table 3:** Phytochemical analysis of the plant extracts from *Combretum albidum* (CA), *Hibiscus acetosella* (HA), *Hibiscus cannabinus* (HC), *Hibiscus furcatus* (HF), *Punica granatum* (PG), and *Tamarindus indica* (TI).

Plant extract	Concentration of secondary metabolites (μg/ml)						
	Phenolic compounds	Terpenoids	Alkaloids	Glycosides	Steroids	Flavonoids	Saponins
CA	5.75 ± 0.14	2.29 ± 0.003	3.46 ± 0.003	3.04 ± 0.16	1.54 ± 0.007	9.26 ± 0.02	1.64 ± 0.10
HA	0.52 ± 0.09	3.04 ± 0.003	2.44 ± 0.005	3.61 ± 0.39	1.52 ± 0.007	9.69 ± 0.25	0.98 ± 0.05
HC	0.33 ± 0.07	6.37 ± 0.013	3.48 ± 0.002	2.87 ± 0.07	1.94 ± 0.002	7.34 ± 0.02	0.58 ± 0.06
HF	0.30 ± 0.07	3.70 ± 0.007	2.44 ± 0.004	4.27 ± 0.38	2.81 ± 0.003	8.12 ± 0.07	1.66 ± 0.16
PG	11.02 ± 0.49	6.67 ± 0.017	1.88 ± 0.007	2.83 ± 0.16	1.05 ± 0.007	5.95 ± 0.05	1.07 ± 0.06
TI	0.19 ± 0.04	1.13 ± 0.013	2.07 ± 0.002	1.67 ± 0.06	0.72 ± 0.005	2.57 ± 0.01	0.56 ± 0.06

### Combinatory Effects of Plant Extracts with Antibiotics against NDM-1 *E. coli*

Combinatory effects were determined by calculating the ΣFIC index for colistin, meropenem, and tetracycline with all six plant extracts. Different concentrations of antibiotics and plant extracts were combined to check for synergistic activity. The observed ΣFIC values when the plant extracts were combined with colistin, meropenem, and tetracycline antibiotics were 0.12–0.31, 0.09–0.31, and 0.12–0.37, respectively. This indicated that the plant extracts have synergistic effects against NDM-1 *E. coli* when combined with all of the three tested antibiotics (ΣFIC ≤ 0.5). *Hibiscus cannabinus* showed consistently lower ΣFIC, and the smallest ΣFIC was observed when it was combined with meropenem (0.09). *Punica granatum* showed consistently higher ΣFIC, and the highest ΣFIC was observed when it was combined with tetracycline (0.37). In terms of reduction in MIC of antibiotics when combined with plant extracts, we have observed between 4- and 16-fold reduction for individual combinations. The highest reduction in MIC against NDM-1 *E. coli* was observed in combinations of colistin with CA and HC; meropenem with HA, HC, and HF; and tetracycline with HC and TI (**Table 4**).

**Table 4:** Combinatory effects of colistin, meropenem, and tetracycline in combination with the extracts of CA, HA, HC, HF, PG, and TI against NDM-1 *E. coli*.

Plant extract	ΣFIC			Fold reduction in MIC		
	Colistin	Meropenem	Tetracycline	Colistin	Meropenem	Tetracycline
CA	0.12	0.19	0.25	16	8	8
HA	0.25	0.12	0.25	8	16	8
HC	0.12	0.09	0.12	16	16	16
HF	0.18	0.12	0.25	8	16	8
PG	0.31	0.31	0.37	4	4	4
TI	0.19	0.25	0.31	16	8	16

### Inhibition of Recombinant NDM-1 Enzyme by the Plant Extracts

The ability of the six plant extracts to inhibit the activity of NDM-1 enzyme was tested *in vitro* using nitrocefin as a chromogenic substrate. The NDM-1 enzyme hydrolyzes the nitrocefin to form a red colored product. Recombinant NDM-1 enzyme activity in the reaction, which contained only nitrocefin in DMSO, was considered 100%. Treatment with the plant extract reduced the activity of NDM-1 enzyme in a time-dependent manner. When treated with 5.12 ng/μl plant extract for 4 h, the highest percentage of enzyme inhibition was observed with HC (77%), followed by CA (67%), PG (59%), HF (57%), TI (53%), and HA (40%). The calculated IC50 in terms of nanograms per microliter was the lowest with HC (0.50), followed by CA (0.73), PG (0.76), HF (0.77), TI (0.78), and HA (1.2).

### Effect of Plant Extracts on Membrane Integrity of NDM-1 *E. coli*

**FIGURE 2:**
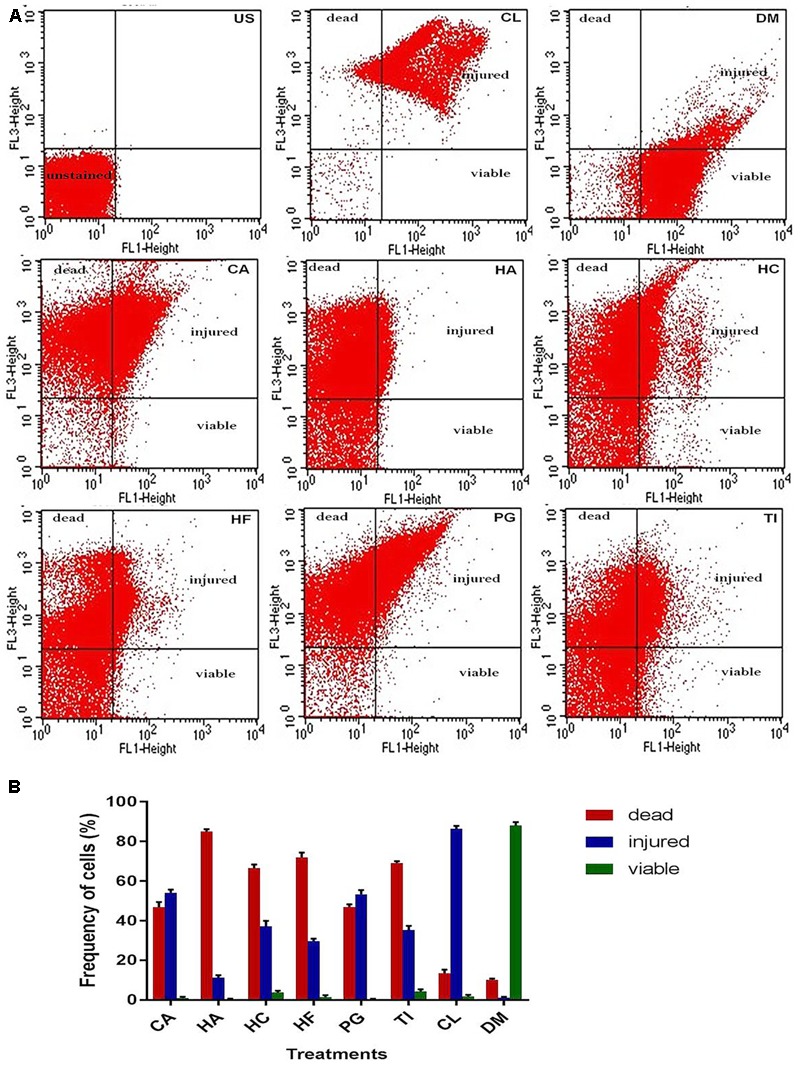
Flow cytometry viability analysis of NDM-1 *E. coli* cells based on SYTO 9 (FL1)/PI (FL3) dot plots **(A)** and estimation of frequency of live, injured, and dead cells **(B)** after the 4 h treatment with 2× MIC of plant extracts from CA, HA, HC, HF, PG, TI, 10 μg colistin (CL, positive control), and 20 μl DMSO (DM, negative control). The quadrants show unstained region (US), PI positive dead region (bacterial cells with irreversibly damaged membranes), SYTO 9 positive live region (bacterial cells with intact plasma membranes), and PI/SYTO 9 double positive injured region (bacterial cells with different degree of disrupted membranes).

**FIGURE 3:**
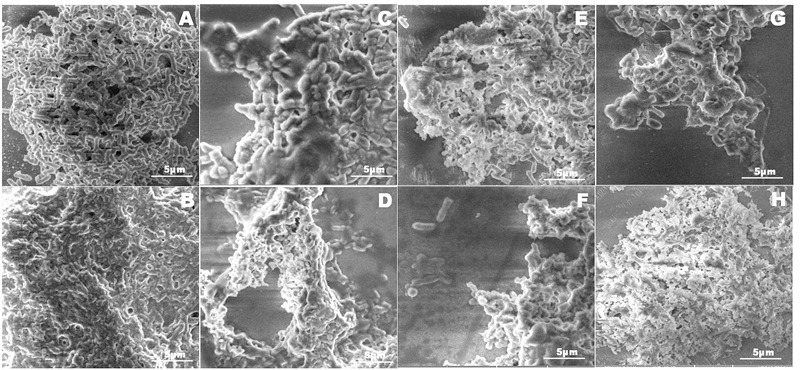
Scanning electron microscope micrograph images of NDM-1 *E. coli* cells that were treated with DMSO **(A)**, colistin **(B)**, and the plant extracts from CA **(C)**, HA **(D)**, HC **(E)**, HF **(F)**, PG **(G)**, and TI **(H)**. Membranes of the bacterial cells treated with DMSO were intact indicating that the cells were alive. Cells treated with colistin **(B)** and the plant extracts **(C–G)** displayed corrugated, wrinkled, shrunken, and deformed surface morphology indicating cell death.

Integrity of the NDM-1 *E. coli* cells after treatment with the plant extracts was studied using the LIVE/DEAD permeability assay and SEM analysis. A large number of NDM-1 *E. coli* cells were found to be dead after the 4 h treatment period, which showed that the plant extracts were highly effective against NDM-1 *E. coli*. All the extracts caused an increase in cell permeability as indicated by PI uptake. Viability of NDM-1 *E. coli* cells was initially 88.3% with DMSO (negative control), which was drastically reduced to as low as 0.3% when treated with plant extracts. This was lower than the viability of the cells treated with colistin, which was 1.8%. The order of plant extracts based on the viability of NDM-1 *E. coli* cells after the 4 h treatment was TI < HC < HF < CA < PG < HA (**Figures 2A,B**). The percentage of cell death ranged between 79.4 and 89.7% (HA and TI, respectively) when treated with the plant extracts. In the SEM analysis, untreated NDM-1 *E. coli* cells displayed smooth and intact cell surfaces, while the cells treated with the plant extracts exhibited corrugated, wrinkled, shrunken, and deformed surface morphologies (**Figure 3**).

## Discussion

Emerging multi-drug resistant bacteria are posing great health risks to human and animals. New Delhi metallo-β-lactamase 1 bacteria were reported to be resistant to most of the current generation of antibiotics, including β-lactam antibiotics (Kumarasamy et al., 2010). The results from the double disk synergy test and sequencing of 16S rRNA and NDM-1 genes confirmed that the strain used in the present study belongs to NDM-1 *E. coli*. This strain was susceptible only to colistin. Colistin was discontinued in the 1970s from clinical use due to its reported nephrotoxicity and neurotoxicity, but it re-emerged as a potent antibiotic to control infections due to NDM-1 bacteria. However, the decision to use colistin and its proper dose needs to be critically evaluated considering the risk and benefits on a case-to-case basis (Betrosian et al., 2008; Lim et al., 2011; Ortwine et al., 2015). Therefore, there is a worldwide attempt to find safe antimicrobial compounds against NDM-1 bacteria. Although certain synthetic compounds and natural compounds from fungi were investigated for this purpose (King et al., 2014), the plant kingdom, which is the main source of drugs, remains unexplored. In the present study, ethanol extracts from the leaves of 240 taxonomically diverse medicinal plant species were screened for antibacterial activities against NDM-1 *E. coli.*

Among the ethanol extracts from the leaves of 12 plants that showed bactericidal activity at 25 mg/ml, the extracts from CA, HA, HC, HF, PG, and TI were found to be promising with MBCs ranging from 5 to 15 mg/ml. Earlier studies have reported that methanol extract from the CA leaves showed antibacterial activity against *Pseudomonas aeruginosa* with an MBC of 4.39 mg/ml (Sahu et al., 2014; Chandar and MohanKumar, 2016), which is lower than the MBC for the ethanol extract against NDM-1 *E. coli* observed in the present study. This species was reported to contain secondary metabolites compounds such as gallic acid and ursolic acid (Kumar et al., 2015), which have been reported to disrupt bacterial cell membranes (Nohynek et al., 2006; Tsuchiya, 2015). There is no report about antibacterial activity for the leaf extract of PG; however, a mixture of pericarp, leaf, and flower extracts was reported to have antibacterial activity against Gram-positive and Gram-negative bacteria (Naz et al., 2007; Dey et al., 2012).

Ethanol extracts from TI leaves have been reported to have antibacterial activity against *E. coli*, Proteus mirabilis, *P. aeruginosa, Salmonella typhii, S. paratyphi, Shigella flexneri*, Staphylococcus aureus, Bacillus subtilis, and Streptococcus pyogenes with MBC between 10 and 20 mg/ml (Doughari, 2006). Ethanol extract from the seeds of TI showed MBC of 125 mg/ml against *E. coli* and *S. aureus*, and 250 mg/ml against *P. aeruginosa* and *B. subtilis* (Nwodo et al., 2011). The authors have not tested concentrations ≤125 mg/ml, which may be the reason for reporting higher MBCs. Methanol extracts from the TI leaves showed antibacterial activity against only one of the 27 tested strains from *E. coli*, Enterobacter cloacae, *K. pneumoniae, P. aeruginosa*, Providencia stuartii, and *E. aerogenes*, probably because lower concentrations ranging from 0.256 to 1.024 mg/ml were tested (Djeussi et al., 2013). Tamarindus indica seed and fruit extracts were effective against several multi drug resistant (MDR) bacteria (Patel et al., 2013; Chowdhury et al., 2013). Flavonoids present in TI leaves caused cell membrane disruption in *E. coli, K. pneumoniae*, methicillin-resistant *Staphylococcus aureus* (MRSA), *S. aureus, P. aeruginosa*, and *B. subtilis* (Gumgumjee et al., 2012). The current study reports the lowest MBC for TI against NDM-1 *E. coli*. Antibacterial activities for the extracts of HA, HC, and HF extracts are reported for the first time.

Leaf extracts from the six selected plants contained phytochemicals such as tannins, terpenoids, alkaloids, glycosides, saponins, and flavonoids. All six plant extracts inhibited the NDM-1 enzyme activity *in vitro*. It may be due to direct enzyme inactivation or through chelation of the enzyme bound zinc ions, which are essential for the catalytic activity (Denny et al., 2002; Wei and Guo, 2014). The flavonoid galangin inhibited MBL from *Stenotrophomonas maltophilia* without metal chelation (Denny et al., 2002). Flavonoids from plant extracts such as 3′,4′-dihydroxylflavone, chrysin, kaempferol, myricetin, and rutin were reported to bind zinc ions at specific sites, and chelate them to form a complex (Kostyuk et al., 2004; Wei and Guo, 2014). Tannins in almond nuts have been reported to show as much as 84% chelation of zinc ions (Karamać, 2009). Therefore, the plant extracts that were identified to be effective against NDM-1 *E. coli* in the present study may have one or more specific phytochemicals, which have the ability to inhibit the NDM-1 enzyme *in vitro*.

Earlier studies using plant extracts have shown that cell membrane damage is one of the mechanisms of action against bacteria (Di Pasqua et al., 2007; Yossa et al., 2012). Cell membrane integrity of various bacterial species after the treatment with different plant extracts was quantitatively assessed using flow cytometry (Kennedy et al., 2011; Saritha et al., 2015). Integrity of the NDM-1 *E. coli* cells after the treatment with the six plant extracts was studied using the LIVE/DEAD permeability assay by staining with red fluorescent PI coupled to green fluorescent SYTO9. Viable bacterial population showed strong green fluorescence and weak red fluorescence while a completely permeabilized population showed weak green fluorescence and strong red fluorescence. In our study, an increase in NDM-1 *E. coli* cell membrane permeability after the treatment with different plant extracts caused 79.4–89.7% cell death. A significant PI uptake by the NDM-1 *E. coli* cells treated with the plant extracts indicated damage to the cell membrane, which was also observed from SEM analysis of the cells. While the SEM images of untreated NDM-1 *E. coli* showed intact cell membranes, the cells treated with the plant extracts were corrugated and disrupted. Several phenolic compounds such as gallic acid, chlorogenic acid, and 3-*p*-*trans*-coumaroyl-2-hydroxyquinic acid have been reported to inhibit bacterial growth by disrupting cell membranes (Nohynek et al., 2006; Wu et al., 2016; Rempe et al., 2017). Similarly, flavonoids such as quercetin and epicatechin gallate have also been shown to disrupt bacterial cell membranes (Xia et al., 2010; da Silva et al., 2014).

Furthermore, although all six plant extracts inhibited NDM-1 activity and increased the membrane permeability of NDM-1 *E. coli*, the actual compound responsible for the action may be different. The most common site of action for plant secondary metabolites is cell membrane; however, the combinatory effects of antibiotics with natural antibacterial agents may help to overcome bacterial resistance through several interesting strategies and delay the recurrence of resistant bacteria (Kao et al., 2010; Wink et al., 2012; dos Santos et al., 2015; Silva et al., 2016). Interestingly, all the plant extracts in combination with colistin, meropenem, and tetracycline reduced the MIC of the antibiotic from 4- to 16-fold. This indicates resistance reversal in NDM-1 *E. coli* due to the compounds present in these plant extracts, which have the ability to inhibit the NDM-1 enzyme and disrupt bacterial cell membranes. The NDM-1 inhibitory phytochemicals in the plant extracts should help overcome enzyme-mediated antibiotic hydrolysis thus rendering the bacteria more susceptible to their actions, as long as such compounds are able to penetrate the cell membrane (Hemaiswarya et al., 2008). The reduction in MIC demonstrated that antibiotic use could be significantly reduced when combined with the plant extracts, indicating the possibility of combination therapy against NDM-1 bacteria.

## Conclusion

By identifying six plants that showed potent antibacterial activity against a NDM-1 *E. coli* strain, the present study demonstrated that plants can be a source of antibacterial compounds against NDM-1 bacteria. Inhibition of NDM-1 enzyme activity and damage to the cell membrane were found to be the possible mechanism of plant extract action against the NDM-1 *E. coli*. Synergistic effects between the antibiotics and plant extracts indicate the possibility of combination therapy against NDM-1 bacteria. One drawback of using crude extracts is that the antimicrobial activity of extracts may result from combinations of compounds, rather than a single compound. If this turns out to be the case, then the antimicrobial properties of individual compounds may actually be less. However, it may also be possible to identify a minor compound with greater potency.

## Author Contributions

MP has supervised the entire project, involved in building the experimental data and interpretation. He also wrote and revised the manuscript, validated the final results, and has approved the final version for publication. BC has designed, performed all the experiments, observed, and analyzed all the experimental data. She has collected all the plants, bacterial samples, and involved in the interpretation of the work. She has also written the manuscript, in an agreement to be accountable for all the investigations, and has approved the final version to be published. RM was involved in designing the experiments that involved extraction from plant samples and conducting them. He has advised for the interpretation of photochemical analysis, edited the manuscript, validated, and approved the publication. KI has also supervised the phytochemical part of the work. He was involved in editing of manuscript and approved for publication. SP was involved in the interpretation of results, editing of manuscript, and approved the final version of the manuscript for publication.

## Conflict of Interest Statement

The authors declare that the research was conducted in the absence of any commercial or financial relationships that could be construed as a potential conflict of interest.
