# Supplementary material for: Inhibition of New Delhi Metallo-β-Lactamase 1 (NDM-1) Producing Escherichia coli IR-6 by Selected Plant Extracts and Their Synergistic Actions with Antibiotics
Source: Front Microbiol. 2017 Aug 22;8:1580. doi: 10.3389/fmicb.2017.01580 (PMC5572277; doi:10.3389/fmicb.2017.01580)
Supplement: Supplementary file 1 [file Table_1.DOCX]

| TableS1: List of plants extracts tested against NDM-1 producing *Escherichia coli* | | | |
| --- | --- | --- | --- |
| Voucher ID | Plant name |  |  |
|  |  | Genus | Family |
| KL046 | *Abrus precatorius* L. | Abrus | Leguminosae |
| TN022 | *Abutilon indicum* (L.) Sweet | Abutilon | Malvaceae |
| TN037 | *Acacia auriculiformis* Benth. | Acacia | Leguminosae |
| TN042 | *Acacia nilotica* (L.) Delile | Acacia | Leguminosae |
| TN079 | *Acalypha indica* L. | Acalypha | Euphorbiaceae |
| TN098 | *Acalypha wilkesiana* Müll.Arg. | Acalypha | Euphorbiaceae |
| TN101 | *Achyranthes aspera* L. | Achyranthes | Amaranthaceae |
| KL055 | *Aegle marmelos* (L.) Corrêa | Aegle | Rutaceae |
| TN119 | *Aerva lanata* (L.) Juss. | Aerva | Amaranthaceae |
| TN043 | *Ageratum conyzoides* (L.) L. | Ageratum | Compositae |
| TN087 | *Alangium salviifolium* (L.f.) Wangerin | Alangium | Cornaceae |
| TN100 | *Albizia saman* (Jacq.) Merr. | Albizia | Leguminosae |
| KL007 | *Albizia lebbeck* (L.) Benth. | Albizia | Leguminosae |
| TN047 | *Alcea rosea* L. | Alcea | Malvaceae |
| TN128 | *Allium sativum* L. | Allium | Amaryllidaceae |
| TN010 | *Allophylus serratus* (Hiern) Kurz | Allophylus | Sapindaceae |
| TN061 | *Alternanthera sessilis* (L.) R.Br. ex DC. | Alternanthera | Amaranthaceae |
| TN050 | *Amaranthus spinosus* L. | Amaranthus | Amaranthaceae |
| TN140 | *Amaranthus viridis* L. | Amaranthus | Amaranthaceae |
| KL036 | *Anamirta cocculus* (L.) Wight & Arn. | Anamirta | Menispermaceae |
| TN071 | *Andrographis paniculata* (Burm.f.) Nees | Andrographis | Acanthaceae |
| TN024 | *Anisomeles indica* (L.) Kuntze | Anisomeles | Lamiaceae |
| TN028 | *Annona reticulata* L. | Annona | Annonaceae |
| KA006 | *Annona squamosa* L. | Annona | Annonaceae |
| TN127 | *Antigonon leptopus* Hook. & Arn. | Antigonon | Polygonaceae |
| TN120 | *Argemone mexicana* L. | Argemone | Papaveraceae |
| KL035 | *Aristolochia indica* L. | Aristolochia | Aristolochiaceae |
| TN089 | *Aristolochia macrophylla* Lam. | Aristolochia | Aristolochiaceae |
| KL026 | *Artemisia nilagirica* (C.B.Clarke) Pamp. | Artemisia | Compositae |
| TN056 | *Asparagus racemosus* Willd. | Asparagus | Asparagaceae |
| TN019 | *Asystasia gangetica* (L.) T.Anderson | Asystasia | Acanthaceae |
| TN163 | *Azadirachta indica* A.Juss. | Azadirachta | Meliaceae |
| TN138 | *Bacopa monnieri* (L.) Wettst. | Bacopa | Plantaginaceae |
| TN032 | *Barleria cristata* L. | Barleria | Acanthaceae |
| TN148 | *Barleria prionitis* L. | Barleria | Acanthaceae |
| TN126 | *Bauhinia tomentosa* L. | Bauhinia | Leguminosae |
| KL011 | *Bixa orellana* L. | Bixa | Bixaceae |
| KL059 | *Blumea obliqua* (L.) Druce | Blumea | Compositae |
| TN033 | *Boerhavia erecta* L. | Boerhavia | Nyctaginaceae |
| TN080 | *Boerhavia diffusa* L. | Boerhavia | Nyctaginaceae |
| TN009 | *Breynia retusa* (Dennst.) Alston | Breynia | Phyllanthaceae |
| TN097 | *Breynia vitis-idaea* (Burm.f.) C.E.C.Fisch. | Breynia | Phyllanthaceae |
| KL008 | *Bridelia stipularis* (L.) Blume | Bridelia | Phyllanthaceae |
| TN005 | *Buchanania axillaris* (Desr.) Ramamoorthy | Buchanania | Anacardiaceae |
| TN038 | *Butea monosperma* (Lam.) Taub. | Butea | Leguminosae |
| KL021 | *Caesalpinia sappan* L. | Caesalpinia | Leguminosae |
| KA008 | *Callistemon lanceolatus* (Sm.) Sweet | Callistemon | Myrtaceae |
| KL004 | *Calophyllum inophyllum* L. | Calophyllum | Clusiaceae |
| TN102 | *Calophyllum calaba* L. | Calophyllum | Clusiaceae |
| TN099 | *Calotropis procera* (Aiton) Dryand. | Calotropis | Apocynaceae |
| TN140 | *Canavalia gladiata* (Jacq.) DC. | Canavalia | Leguminosae |
| TN041 | *Capparis decidua* (Forssk.) Edgew. | Capparis | Capparaceae |
| TN152 | *Capsicum annuum* L. | Capsicum | Solanaceae |
| KL042 | *Carallia brachiata* (Lour.) Merr. | Carallia | Rhizophoraceae |
| TN035 | *Cardiospermum halicacabum*L. | Cardiospermum | Sapindaceae |
| TN147 | *Carica papaya* L. | Carica | Caricaceae |
| TN125 | *Carissa spinarum* L. | Carissa | Apocynaceae |
| TN039 | *Cascabela thevetia*(L.) Lippold | Cascabela | Apocynaceae |
| KA006 | *Cayratia pedata* (Lam.) Gagnep. | Cayratia | Vitaceae |
| KL009 | *Celastrus paniculatus* Willd. | Celastrus | Celastraceae |
| TN118 | *Centella asiatica* (L.) Urb. | Centella | Apiaceae |
| TN070 | *Cerbera manghas* L. | Cerbera | Apocynaceae |
| KL018 | *Cinnamomum tamala* (Buch.-Ham.) T.Nees & Eberm. | Cinnamomum | Lauraceae |
| TN096 | *Cinnamomum zeylanicum* Blume | Cinnamomum | Lauraceae |
| TN103 | *Cissus quadrangularis* L. | Cissus | Vitaceae |
| TN054 | *Cissus vitiginea* L. | Cissus | Vitaceae |
| TN137 | *Cleome viscosa* L. | Cleome | Cleomaceae |
| TN141 | *Cleome gynandra* L. | Cleome | Cleomaceae |
| KL058 | *Cleome rutidosperma* DC. | Cleome | Cleomaceae |
| KL031 | *Clerodendrum paniculatum* L. | Clerodendrum | Lamiaceae |
| TN157 | *Clerodendrum phlomidis* L.f. | Clerodendrum | Lamiaceae |
| KL043 | *Clitoria ternatea* L. | Clitoria | Leguminosae |
| KL054 | *Coccinia grandis* (L.) Voigt | Coccinia | Cucurbitaceae |
| TN013 | *Cocculus hirsutus* (L.) W.Theob. | Cocculus | Menispermaceae |
| TN012 | *Coldenia procumbens* L. | Coldenia | Boraginaceae |
| TN003 | *Combretum albidum* G. Don | Combretum | Combretaceae |
| TN132 | *Combretum indicum* (L.) DeFilipps | Combretum | Combretaceae |
| TN048 | *Cordia dichotoma* G.Forst. | Cordia | Boraginaceae |
| TN151 | *Coriandrum sativum* L. | Coriandrum | Apiaceae |
| TN158 | *Costus speciosus* (J. Koenig) Sm. | Costus | Costaceae |
| TN124 | *Crateva religiosa* G.Forst. | Crateva | Capparaceae |
| KL023 | *Crotalaria pallida* Aiton | Crotalaria | Leguminosae |
| TN060 | *Crotalaria verrucosa* L. | Crotalaria | Leguminosae |
| TN117 | *Croton bonplandianus* Baill. | Croton | Euphorbiaceae |
| KL025 | *Croton tiglium* L. | Croton | Euphorbiaceae |
| TN075 | *Cucurbita maxima* Duchesne | Curcurbita | Cucurbitaceae |
| TN081 | *Curcuma longa* L. | Curcuma | Zingiberaceae |
| TN026 | *Cuscuta reflexa*Roxb. | Cuscuta | Convolvulaceae |
| KL032 | *Cymbopogon citratus* (DC.) Stapf | Cymbopogon | Poaceae |
| TN004 | *Cyperus rotundus* L. | Cyperus | Cyperaceae |
| KL040 | *Dalbergia latifolia* Roxb. | Dalbergia | Leguminosae |
| TN090 | *Datura metel* L. | Datura | Solanaceae |
| TN104 | *Desmodium gangeticum* (L.) DC. | Desmodium | Leguminosae |
| TN025 | *Desmodium triflorum* (L.) DC. | Desmodium | Leguminosae |
| TN014 | *Dichrostachys cinerea* (L.) Wight & Arn. | Dichrostachys | Leguminosae |
| TN006 | *Diospyros ferrea* (Willd.) Bakh. | Diospyros | Ebenaceae |
| TN008 | *Diospyros melanoxylon* Roxb. | Diospyros | Ebenaceae |
| TN136 | *Dodonaea viscosa* (L.) Jacq. | Dodonaea | Sapindaceae |
| TN040 | *Dregea volubilis* (L.f.) Benth. ex Hook.f. | Dregea | Apocynaceae |
| KL028 | *Elaeocarpus serratus* L. | Elaeocarpus | Elaeocarpaceae |
| KL034 | *Elettaria cardamomum* (L.) Maton | Elettaria | Zingiberaceae |
| KL016 | *Embelia ribes* Burm.f. | Embelia | Primulaceae |
| TN016 | *Erythrina variegata* L. | Erythrina | Leguminosae |
| TN142 | *Eucalyptus tereticornis* Sm. | Eucalyptus | Myrtaceae |
| TN123 | *Eupatorium odoratum* L. | Eupatorium | Asteraceae |
| TN116 | *Euphorbia antiquorum* L. | Euphorbia | Euphorbiaceae |
| TN076 | *Euphorbia heterophylla* L. | Euphorbia | Euphorbiaceae |
| TN095 | *Euphorbia hirta* L. | Euphorbia | Euphorbiaceae |
| TN052 | *Evolvulus alsinoides* (L.) L. | Evolvulus | Convolvulaceae |
| TN105 | *Ficus racemosa* L. | Ficus | Moraceae |
| KL027 | *Ficus longifolia* Schott | Ficus | Moraceae |
| TN017 | *Flacourtia indica* (Burm.f.) Merr. | Flacourtia | Salicaceae |
| KL042 | *Garcinia mangostana* L. | Garcinia | Clusiaceae |
| TN129 | *Gleichenia linearis* (Burm. f.) C.B. Clarke | Gleichenia | Gleicheniaceae |
| KA007 | *Glinus oppositifolius* (L.) Aug.DC. | Glinus | Molluginaceae |
| TN162 | *Gliricidia sepium* (Jacq.) Walp. | Gliricidia | Leguminosae |
| TN159 | *Gloriosa superba* L. | Gloriosa | Colchicaceae |
| KL024 | *Glycosmis pentaphylla* (Retz.) DC. | Glycosmis | Rutaceae |
| TN146 | *Grevillea robusta* A.Cunn. ex R.Br. | Grevillea | Proteaceae |
| KL048 | *Helicteres isora* L. | Helicteres | Malvaceae |
| TN160 | *Heliotropium indicum* L. | Heliotropium | Boraginaceae |
| KL044 | *Hevea brasiliensis* (Willd. ex A.Juss.) Müll.Arg. | Hevea | Euphorbiaceae |
| KL051 | *Hibiscus furcatus* Willd. | Hibiscus | Malvaceae |
| TN031 | *Hibiscus tiliaceus* L. | Hibiscus | Malvaceae |
| TN030 | *Hibiscus acetosella* Welw. ex Hiern | Hibiscus | Malvaceae |
| KA003 | *Hibiscus cannabinus* L. | Hibiscus | Malvaceae |
| TN153 | *Hibiscus rosa-sinensis* L. | Hibiscus | Malvaceae |
| TN130 | *Hibiscus vitifolius* L. | Hibiscus | Malvaceae |
| KL038 | *Holarrhena antidysenterica* (L.) Wall. ex A. DC. | Holarrhena | Apocynaceae |
| TN049 | *Hybanthus enneaspermus* (L.) F.Muell. | Hybanthus | Violaceae |
| TN115 | *Hydnocarpus alpina* Wight | Hydnocarpus | Achariaceae |
| KA001 | *Hygrophila auriculata* (Schumach.) Heine | Hygrophila | Acanthaceae |
| TN067 | *Hyptis suaveolens* (L.) Poit. | Hyptis | Lamiaceae |
| KA004 | *Ichnocarpus frutescens* (L.) W.T.Aiton | Ichnocarpus | Apocynaceae |
| TN034 | *Ipomoea obscura* (L.) Ker Gawl. | Ipomoea | Convolvulaceae |
| TN082 | *Ipomoea palmata* Forssk. | Ipomoea | Convolvulaceae |
| TN011 | *Ipomoea aquatica* Forssk. | Ipomoea | Convolvulaceae |
| TN059 | *Jatropha curcas* L. | Jatropha | Euphorbiaceae |
| TN015 | *Justicia adhatoda* L. | Justicia | Acanthaceae |
| TN106 | *Lantana camara* L. | Lantana | Verbenaceae |
| TN145 | *Lawsonia inermis* L. | Lawsonia | Lythraceae |
| KL041 | *Leea indica* (Burm. f.) Merr. | Leea | Vitaceae |
| KL053 | *Leucas aspera* (Willd.) Link | Leucas | Lamiaceae |
| TN065 | *Limnophila indica* (L.) Druce | Limnophila | Plantaginaceae |
| KA002 | *Luffa cylindrica* (L.) M.Roem. | Luffa | Cucurbitaceae |
| TN135 | *Maba buxifolia* (Rottb.) Pers. | Maba | Ebenaceae |
| KL006 | *Madhuca longifolia* (J.Koenig ex L.) J.F.Macbr. | Madhuca | Sapotaceae |
| TN078 | *Melastoma malabathricum* L. | Melastoma | Melastomataceae |
| TN046 | *Mentha piperita* L. | Mentha | Lamiaceae |
| KA007 | *Merremia emarginata* (Burm. f.) Hallier f. | Merremia | Convolvulaceae |
| KL001 | *Mesua ferrea* L. | Mesua | Calophyllaceae |
| KL039 | *Michelia champaca* L. | Michelia | Magnoliaceae |
| KL050 | *Mikania micrantha* Kunth | Mikania | Compositae |
| TN144 | *Mirabilis jalapa* L. | Mirabilis | Nyctaginaceae |
| TN122 | *Mollugo nudicaulis* Lam. | Mollugo | Molluginaceae |
| TN114 | *Morinda tinctoria* Roxb. | Morinda | Rubiaceae |
| TN074 | *Moringa oleifera* Lam. | Moringa | Moringaceae |
| TN083 | *Mukia maderaspatana* (L.) M.Roem. | Mukia | Cucurbitaceae |
| TN058 | *Murraya koenigii* (L.) Spreng. | Murraya | Rutaceae |
| TN094 | *Musa paradisiaca* L. | Musa | Musaceae |
| KL029 | *Neolamarckia cadamba* (Roxb.) Bosser | Neolamarckia | Rubiaceae |
| TN107 | *Ocimum sanctum* L. | Ocimum | Lamiaceae |
| TN131 | *Ocimum gratissimum* L. | Ocimum | Lamiaceae |
| KA011 | *Ocimum tenuiflorum* L. | Ocimum | Lamiaceae |
| KL013 | *Olea dioica* Roxb. | Olea | Oleaceae |
| KL020 | *Oroxylum indicum* (L.) Kurz | Oroxylum | Bignoniaceae |
| TN029 | *Oxalis corniculata* L. | Oxalis | Oxalidaceae |
| TN164 | *Oxystelma esculentum* (L. f.) Sm. | Oxystelma | Apocynaceae |
| TN064 | *Parthenium hysterophorus* L. | Parthenium | Compositae |
| TN150 | *Passiflora foetida* L. | Passiflora | Passifloraceae |
| TN161 | *Phyllanthus emblica* L. | Phyllanthus | Phyllanthaceae |
| TN023 | *Phyllanthus amarus* Schumach. & Thonn. | Phyllanthus | Phyllanthaceae |
| TN001 | *Phyllanthus maderaspatensis* L. | Phyllanthus | Phyllanthaceae |
| KL057 | *Phyllanthus niruri* L. | Phyllanthus | Phyllanthaceae |
| TN139 | *Physalis minima* L | Physalis | Solanaceae |
| KL060 | *Piper argyrophyllum* Miq. | Piper | Piperaceae |
| KL062 | *Piper betle* L. | Piper | Piperaceae |
| KL061 | *Piper nigrum* L. | Piper | Piperaceae |
| TN027 | *Plumbago zeylanica* L. | Plumbago | Plumbaginaceae |
| TN156 | *Plumeria rubra* L. | Plumeria | Apocynaceae |
| TN020 | *Polyalthia longifolia* (Sonn.) Thwaites | Polyalthia | Annonaceae |
| KA009 | *Portulaca oleracea* L. | Portulaca | Portulacaceae |
| KL045 | *Pterocarpus marsupium* Roxb. | Pterocarpus | Leguminosae |
| TN057 | *Pterolobium hexapetalum* (Roth) Santapau & Wagh | Pterolobium | Leguminosae |
| KL003 | *Pterospermum canescens* Roxb. | Pterospermum | Malvaceae |
| KL012 | *Pterospermum rubiginosum* Heyne | Pterospermum | Malvaceae |
| TN149 | *Pterospermum acerifolium* (L.) Willd. | Pterospermum | Malvaceae |
| KL056 | *Punica granatum* L. | Punica | Lythraceae |
| KL030 | *Ruta graveolens* L. | Ruta | Rutaceae |
| KL017 | *Saraca asoca* (Roxb.) Willd. | Saraca | Leguminosae |
| TN113 | *Scoparia dulcis* L. | Scoparia | Plantaginaceae |
| TN062 | *Scutia myrtina* (Burm.f.) Kurz | Scutia | Rhamnaceae |
| TN068 | *Senna alata* (L.) Roxb. | Senna | Leguminosae |
| TN084 | *Senna auriculata* (L.) Roxb. | Senna | Leguminosae |
| TN091 | *Senna siamea* (Lam.) H.S.Irwin & Barneby | Senna | Leguminosae |
| TN109 | *Sesbania grandiflora* (L.) Pers. | Sesuvium | Aizoaceae |
| KA010 | *Sesuvium portulacastrum* (L.) L. | Sesbania | Leguminosae |
| TN122 | *Sida acuta* Burm.f. | Sida | Malvaceae |
| TN021 | *Sida cordifolia* L. | Sida | Malvaceae |
| TN051 | *Simarouba glauca* DC. | Simarouba | Simaroubaceae |
| TN073 | *Solanum nigrum* L. | Solanum | Solanaceae |
| TN002 | *Solanum trilobatum* L. | Solanum | Solanaceae |
| TN022 | *Solanum surattense* Burm. f. | Solanum | Solanaceae |
| TN085 | *Solanum virginianum* L. | Solanum | Solanaceae |
| TN112 | *Spathodea campanulata* P.Beauv. | Spathodea | Bignoniaceae |
| KL064 | *Sphagneticola calendulacea* (L.) Pruski | Sphagneticola | Compositae |
| TN134 | *Stachytarpheta indica* (L.) Vahl | Stachytarpheta | Verbenaceae |
| KL047 | *Stereospermum chelonoides* (L.f.) DC. | Stereospermum | Bignoniaceae |
| KL019 | *Strobilanthes ciliatus* Wall. ex Nees | Strobilanthes | Acanthaceae |
| KA005 | *Swietenia macrophylla* King | Swietenia | Meliaceae |
| KL015 | *Symplocos cochinchinensis* (Lour.) S. Moore | Symplocos | Symplocaceae |
| TN155 | *Syzygium cumini* (L.) Skeels | Syzygium | Myrtaceae |
| KL010 | *Syzygium stocksii* (Duthie) Gamble | Syzygium | Myrtaceae |
| KL005 | *Tamarindus indica* L. | Tamarindus | Leguminosae |
| TN154 | *Tarenna asiatica* (L.) Kuntze ex K.Schum. | Tarenna | Rubiaceae |
| TN092 | *Tephrosia purpurea*(L.) Pers. | Tephrosia | Leguminosae |
| TN143 | *Terminalia arjuna* (Roxb. ex DC.) Wight & Arn. | Terminalia | Combretaceae |
| TN069 | *Terminalia chebula* Retz. | Terminalia | Combretaceae |
| KL037 | *Terminalia paniculata* Roth | Terminalia | Combretaceae |
| TN055 | *Terminalia bellirica* (Gaertn.) Roxb. | Terminalia | Combretaceae |
| KL063 | *Terminalia catappa* L. | Terminalia | Combretaceae |
| KL052 | *Terminalia muelleri* Benth. | Terminalia | Combretaceae |
| TN110 | *Tiliacora racemosa* Colebr. | Tiliacora | Menispermaceae |
| TN066 | *Tinospora cordifolia* (Willd.) Miers ex Hook. f. & Thomson | Tinospora | Menispermaceae |
| KL033 | *Tinospora sinensis* (Lour.) Merr. | Tinospora | Menispermaceae |
| TN133 | *Tragia involucrata* L. | Tragia | Euphorbiaceae |
| TN121 | *Tridax procumbens* (L.) L. | Tridax | Compositae |
| KL049 | *Triumfetta rhomboidea* Jacq. | Triumfetta | Malvaceae |
| KL002 | *Vateria indica* L. | Vateria | Dipterocarpaceae |
| TN072 | *Vernonia cinerea* (L.) Less. | Vernonia | Compositae |
| KL022 | *Vernonia arborea* Buch.-Ham. | Vernonia | Compositae |
| KL014 | *Vitex altissima* L.f. | Vitex | Lamiaceae |
| TN086 | *Vitex negundo* L. | Vitex | Lamiaceae |
| TN045 | *Xanthium strumarium* L. | Xanthium | Compositae |
| TN111 | *Ximenia americana* L. | Ximenia | Olacaceae |
| TN053 | *Zaleya decandra* (L.) Burm.f. | Zaleya | Aizoaceae |
| TN093 | *Zingiber officinale* Roscoe | Zingiber | Zingiberaceae |
| TN063 | *Ziziphus mauritiana* Lam. | Ziziphus | Rhamnaceae |
| TN036 | *Ziziphus jujuba* Mill. | Ziziphus | Rhamnaceae |
| TN007 | *Ziziphus xylopyrus* (Retz.) Willd. | Ziziphus | Rhamnaceae |
